# The actin cytoskeleton and small G protein RhoA are not involved in flow-dependent activation of ENaC

**DOI:** 10.1186/1756-0500-3-210

**Published:** 2010-07-27

**Authors:** Alexey V Karpushev, Daria V Ilatovskaya, Alexander Staruschenko

**Affiliations:** 1 Department of Physiology, Medical College of Wisconsin, 8701 Watertown Plank Rd., Milwaukee, WI 53226, USA; 2 Kidney Disease Center, Medical College of Wisconsin, 8701 Watertown Plank Rd., Milwaukee, WI 53226, USA; 3 Institute of Cytology, Russian Academy of Sciences, St. Petersburg, 194064, Russian Federation

## Abstract

**Background:**

Epithelial cells are exposed to a variety of mechanical stimuli. Epithelial Na^+ ^channels (ENaC) mediate sodium transport across apical membranes of epithelial cells that line the distal nephron, airway and alveoli, and distal colon. Early investigations into stretch sensitivity of ENaC were controversial. However, recent studies are supportive of ENaC's mechanosensitivity. This work studied whether flow-dependent activation of ENaC is modulated by changes in the state of the actin cytoskeleton and whether small GTPase RhoA is involved in flow-mediated increase of ENaC activity.

**Findings:**

Pretreatment with Cytochalasin D and Latrunculin B for 20 min and 1-2 hrs to disassemble F-actin had no effect on flow-mediated increase of amiloride-sensitive current. Overexpression of ENaC with constitutively active (G14V) or dominant negative (T19N) RhoA similarly had no effect on flow-dependent activation of ENaC activity. In addition, we did not observe changes when we inhibited Rho-kinase with Y27632.

**Conclusions:**

Our results suggest that the flow-dependent activation of ENaC is not influenced by small GTPase RhoA and modifications in the actin cytoskeleton.

## Introduction

The long term control of blood pressure involves Na^+ ^homeostasis through the precise regulation of the Epithelial Na^+ ^Channel (ENaC) in the aldosterone-sensitive distal nephron [1,2]. The rate of Na^+ ^absorption varies widely in response to conditions of Na^+ ^deprivation and Na^+ ^excess. The physiological relevance of the mechanosensitivity of ENaC becomes clearly apparent in the mammalian cortical collecting ducts (CCDs), a nephron segment subject to continuous variations in rates of tubular flow. Flow rates within the distal nephron, including CCDs, increase in response to expansion of the extracellular fluid volume or administration of diuretics and fall in response to volume depletion [3]. Palmer and Frindt studied whether mechanical perturbations could influence ENaC kinetics or gating mode in freshly isolated rat CCDs. In most cases negative pressure applied to the patch clamp pipette had no effect on channel behaviour. However they also observed a rapid and reversible increase in channel open probability (*P*_o_) in 6 out of 22 patches [4]. In one experiment, there was a reversible decrease. They proposed that the variability in the response could reflect differences in the mechanical deformations of the apical membrane within the tip of the pipette [4]. Later Awayda and Subramanyam proposed that rENaC is not directly mechanosensitive when overexpressed in oocytes [5]. However, recent data are mostly supportive of ENaC's mechanosensitivity. Thus it was shown that the response to flow reflects a rapid increase in the open probability of ENaC [6-11]. Moreover, it was proposed that aldosterone modifies the osmotic stress-induced regulation of ENaC trafficking [10].

While there are many studies demonstrating flow-mediated regulation of ENaC, mechanisms underlying this activation of ENaC are not clear yet. Biological membranes are capable of deforming in response to external stresses or through association with the cytoskeleton. Cytoskeletal elements are evidently an important part of ion transport regulation in epithelia. Mechanosensitive channels in various eukaryotic cells are thought to be functionally and structurally coupled to the cortical cytoskeleton. For instance, we have shown previously that the F-actin disassembly resulted in a reduction of the amplitude of stretch-activated currents in human leukaemia K562 cells [12]. Achard et al. [13] demonstrated in human B lymphocytes that a modest increase in the hydrostatic pressure of the solution bathing the cells activated amiloride-sensitive sodium channels, a response requiring an intact cytoskeleton. Inhibition of microtubule polymerization with colchicine prevented stretch-induced activation of Na^+ ^channels [13]. Moreover, it was recently shown that mechanical stimulation of actin stress fibers can activate mechanosensitive channels in HUVEC cells [14].

Actin cytoskeleton and microtubules play an important role in the regulation of membrane transport processes in epithelia [15-19]. We found recently that the cytoskeleton is necessary for small G protein mediated increase of ENaC activity [20]. Acute destroying of the actin cytoskeleton with Cytochalasin D increases ENaC open probability similar to flow-dependent effect on ENaC activity. Small G proteins, molecular switches that control the activity of cellular and membrane proteins, regulate a wide variety of cell functions. As we have shown previously, RhoA activates ENaC via Rho-kinase and subsequent activation of PI(4)P 5-kinase with concomitant increases in PI(4,5)P_2 _levels and promotes channel insertion into the plasma membrane [21-23].

The present study was designed to investigate a hypothesis that the mechanical activation mediated by the cytoskeleton is of considerable physiological relevance and that this ENaC feature could therefore represent a novel nonhormonal regulatory mechanism responsible for flow-mediated activation of ENaC. Moreover, we feel that small G proteins may play a key role in mechanosensitive activation of the ENaC channel and flow-dependent regulation of Na^+ ^transport in the distal nephron. Experiments in this study examined these hypotheses. However, we find that the actin cytoskeleton and small G protein RhoA are not required for flow-mediated activation of ENaC.

## Results

**Figure 1:**
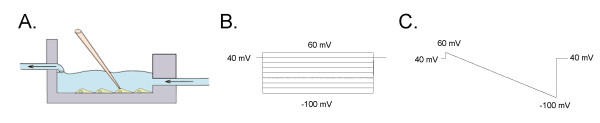
**Experimental setup and protocols**. (A) Experimental setup used to apply flow stimulation. Voltage steps (B) and ramp (C) protocols used to measure amiloride sensitive current in CHO cells.

**Figure 2:**
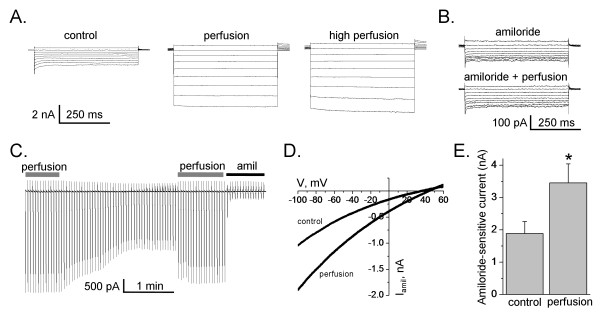
**Flow-dependent activation of ENaC**. (A) Typical macroscopic currents elicited by voltage steps from 60 to -100 mV in voltage clamped CHO cells before and after flow stimulation (low level - middle recording, and high level - right). (B) Current traces were recorded before and after flow stimulation when cells were treated with amiloride (10 μM). (C) The current through ENaC elicited by voltage ramps (60 to -100 mV from a holding potential of 40 mV) applied every 3 s with and without perfusion. Amiloride 10 μM applied at the end of the experiment. (D) Representative current-voltage relation of macroscopic, amiloride-sensitive ENaC currents in CHO cells before (control) and after flow stimulation (perfusion). (E) Summary graph of amiloride-sensitive current density at -80 mV for CHO cells expressing mENaC in control conditions and after flow stimulation. Values are means ± SE of 18 experiments.

To investigate flow-dependent activation of ENaC activity, we reconstituted the channel in Chinese hamster ovary (CHO) cells. mENaC was reconstituted by co-expressing α-, β- and γ-channel subunits. Flow-dependent activation of ENaC activity was induced by activation of bath perfusion surrounding the cells. Amiloride-sensitive current was normalized to the basal activity in the absence of perfusion. Figure 1 shows an experimental setup and protocols used to measure amiloride sensitive whole cell currents in CHO cells. Different experimental systems have their own limitations. Initially we tested the effects of flow stimulation on the whole-cell currents when ENaC was expressed in CHO cells. As shown in Figure 2 flow significantly increases amiloride sensitive current in CHO cells over-expressed with all three ENaC subunits. Figure 2A presents the macroscopic currents from representative whole cell experiments elicited by a set of test pulses stepping from a holding potential of 40 mV to 60 mV to -100 mV (see protocol in Figure 1B). As reported previously, CHO cells contain no endogenous, amiloride-sensitive ENaC current [24]. Currents before (left) and after (middle - low perfusion, and right - high perfusion) flow stimulation are shown. High and low perfusion rates were obtained by increasing the amount of solution that flows through the bath chamber. The high perfusion was the rate threshold beyond that no further increase in whole cell current was observed. In our experiments the effect of flow on the whole cell currents was only slightly progressive (Figure 2A). Similarly, stretch-activation of amiloride-sensitive Na^+ ^channels in human B lymphocytes occurred once a threshold value of hydrostatic pressure was reached. On reaching the threshold, the currents increased maximally, and there was no further increase in current amplitude by further elevation of hydrostatic pressure [13]. Awayda and Subramanyam have shown previously that ENaC whole-cell currents were insensitive to osmotic cell swelling when rENaC was over-expressed in oocytes [5]. They also observed that amiloride-insensitive currents were mediated by cell swelling. Thus, we have tested an effect of flow when cells were pretreated with amiloride. However flow had no effect when amiloride was included in the bath solution (Figure 2B). The finding that the flow-activated current was sensitive to amiloride and that there was no flow-mediated current in untransfected cells (data not shown) clearly indicates that ENaC is activated by flow and that flow activation is not a secondary effect of the experimental setup. Figure 2C shows current through ENaC in the presence and absence of perfusion elicited by a train of voltage ramps applied every 3 s over the course of several minutes. As the perfusion was stopped to maintain a constant bath level, ENaC activity decreased over time indicating that flow-stimulation was necessary to enhance channel activity. Flow-induced activation of ENaC was fast and completely reversible as shown in Figure 2C. The effect of flow on the current was sustained as long as perfusion remained elevated and was immediately reversed (Figure 2C). Figure 2D shows the current-voltage (I-V) relations for macroscopic ENaC current in the control conditions and after flow-dependent activation. As summarized in Figure 2E, perfusion significantly increased ENaC activity from 1.88 ± 0.37 to 3.45 ± 0.59 nA (n = 18).

To estimate whether the flow-dependent activation of ENaC activity is mediated by the actin cytoskeleton, we pretreated CHO cell with Cytochalasin D (CyD) and Latrunculin B (LatB), well known unrelated inhibitors of actin polymerization. CytD binds to the barbed, fast growing plus ends of microfilaments, which then blocks both the assembly and disassembly of individual actin monomers from the bound end [12,25]. In contrast, LatB binds actin monomers and prevents them from polymerizing [26]. We and others have shown earlier that acute application of CytD induced rapid increase in ENaC activity via affecting channel open probability (*Po*). In contrast, long pretreatment with CytD significantly decreased ENaC activity [15,20]. Surprisingly, LatB has no effect on ENaC activity (data not shown). We suggest that as these two reagents have a completely different mechanism leading to disassembly of action cytoskeleton it is more likely that namely actin assembly modulation is responsible for cytoskeleton-dependent mechanism of ENaC regulation. However this exciting finding needs further investigation.

**Figure 3:**
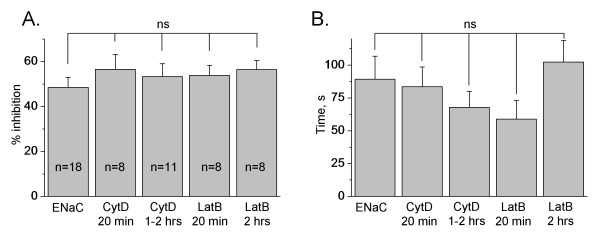
**Destroying of the actin cytoskeleton does not affect flow-mediated activation of ENaC**. (A) Summary graph of inhibition extent of amiloride-sensitive current. The peak response of the whole-cell amiloride-sensitive current at basal conditions was normalized to the flow-stimulated currents. All three mENaC subunits were overexpressed in CHO cells and ENaC activity recorded either with or without perfusion. CHO cells were not treated or pretreated with Cytochalasin D (CytD, 10 μg/ml) or Latrunculin B (LatB, 5 μM) for 20 min and 1-2 hrs, respectively. The number of observations for each group is shown. (B) Summary graph of the time constants describing the rate of ENaC inhibition following switching off flow stimulation of ENaC activity in CHO cells not treated and pretreated with CytD or LatB as presented in (A).

For experiments with inhibitors of the actin cytoskeleton, CHO cells were initially flow-stimulated and ENaC activity was measured before and after the perfusion was eliminated. However, neither 20 min nor 1-2 hrs treatment with CytD or LatB changed the extent of inhibition of flow-stimulated amiloride-sensitive current (Figure 3A). Furthermore, we tested whether destroying of the actin cytoskeleton has an effect on the rate of ENaC inhibition. Time constants are calculated as the time period needed for the current to decrease by 50% after the flow is switched off compared to the current when flow is activated. The time constants describing ENaC inhibition also did not change when the actin cytoskeleton was destroyed (Figure 3B).

**Figure 4:**
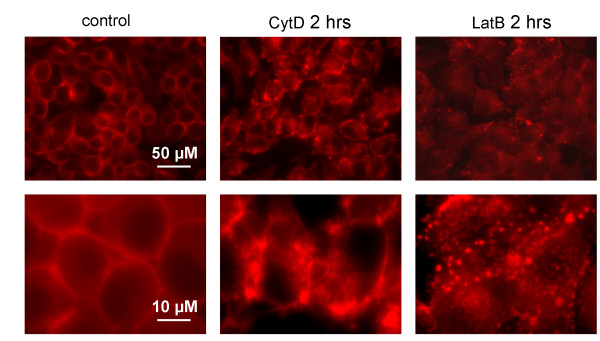
**CytD and LatB destroy the actin cytoskeleton in CHO cells**. Images of CHO cells stained with 2 μM rhodamine-phallodin at 60 × before (left) and after treatment with 20 μM CytD (middle) and with 5 μM LatB (right). Scale bars are shown. The top and bottom rows show expanded and close-up images.

To demonstrate that treatment with CytD and LatB modulated the actin cytoskeleton we performed fluorescence staining of CHO cells with rhodamine-phalloidin. CHO cells were incubated with CytD (20 μM; 2 hrs) and with LatB (5 μM; 2 hrs) and then stained to visualize changes of the actin cytoskeleton state of a cell. Fluorescence microscopy showed that treatment of CHO cells with CytD and LatB caused disruption of the actin cytoskeleton. Untreated cells exhibit typical cortical and filamentous actin staining that becomes depolymerized with the addition of 20 μM CytD or 5 μM LatB (Figure 4).

**Figure 5:**
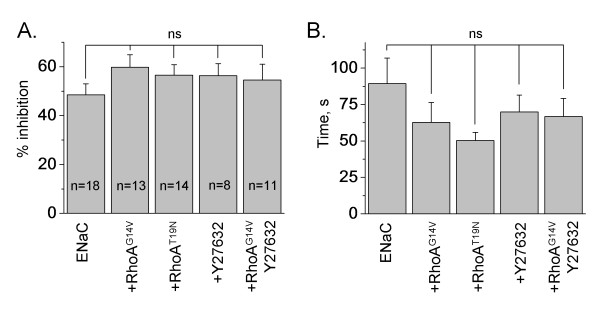
**RhoA does not affect flow-mediated activation of ENaC**. (A, B) Summary graph of an extent (A) and half-time (B) ENaC inhibition after switching off a perfusion. CHO cells expressing either mENaC alone or coexpressed with constitutively active (G14V) or dominant negative (T19N) RhoA in the absence and presence of treatment with Y27632 (1 μM; 1 hr). The number of observations for each group is shown. All other conditions are the same as in Figure 3.

To investigate the involvement of small G protein RhoA in the flow-dependent increase of ENaC activity, we co-expressed ENaC subunits with constitutively active (G14V) and dominant negative (T19N) RhoA. As we have shown previously, wild type and mutant RhoA were over-expressed in CHO cells and modulated ENaC activity [21-23]. In addition, we pretreated CHO cells either expressing ENaC alone or co-expressed with RhoA^G14V ^with Rho-kinase inhibitor (Y27632; 1 μM; 1 hr). Rho-kinase is a downstream effector of RhoA with respect to ENaC [23]. However the extent of inhibition of flow-mediated amiloride-sensitive current was similar in all studied groups (Figure 5A). Also, the time constants describing ENaC inhibition did not change (Figure 5B). We conclude that the actin cytoskeleton and small G protein RhoA are not involved in flow-dependent activation of ENaC.

## Discussion

The epithelial Na^+ ^channel is an essential channel responsible for Na^+ ^reabsorption. Laminar shear stress induced by fluid flow activates ENaC activity *in vivo *and *in vitro*. Different studies indicate that ENaC responds to mechanical forces such as laminar shear stress, osmotic stress and hydrostatic pressure [4-9,13,27-31]. Thus, current investigations established that ENaC is most likely a mechanosensitive ion channel [27,30,32,33]. Shear force-induced ENaC activation is mediated by an increase in *Po *[7,8,34] and is not affected by changes in [Ca^2+^] or membrane trafficking [31]. Moreover, it was shown that the flow-dependent activation of ENaC is not influenced by modifications in the intrinsic properties of the plasma membrane. Flow-dependent activation of ENaC is not affected by the modifications induced by temperature and the content of membrane cholesterol [9]. Trypsin, a protease affecting ENaC *Po*, also did not change ratios of the shear stress-induced effects [8,31]. Fronius and Clauss proposed that large extracellular loops of ENaC play a major role in this mechanical activation [30]. According to this topology, extracellular loops of the channel can be described as mechanosensors oriented into lumen of the CCD. It has been supposed that cystein rich domains of the extracellular loops localized on every ENaC subunit can be involved into modulation of the channel's activity [30]. Furthermore it was shown that ENaCs with deletions of the COOH-terminal domains of the three subunits are still activated by laminar shear stress when expressed in *X. laevis *oocytes [7]. Carattino et al. have proposed that spatial and temporal variations of flow rate in renal tubules produce a deformation of a mechanical sensor that is transduced to the channel gate. Work performed as a result of shear distortion of mechanosensitive structures facilitate conformational changes that eventually gate the channel [34]. Similar to other ion channels, ENaC activity is regulated, in part, by the actin cytoskeleton. Besides, the cytoskeleton is an established target for small G proteins signaling, and particularly RhoA. In current study we attempted to address the role of the actin cytoskeleton and small GTPases in the mechanical activation of ENaC. However, despite mostly negative findings were established, these results provide new important insights into the control of ENaC.

Sipos et al. recently shown that mechanosensitive connexin 30 hemichannels have an integral role in pressure natriuresis by releasing ATP into the tubular fluid, which inhibits salt and water reabsorption [35]. Apical release of ATP can activate P2Y_2 _receptors in the ASDN and inhibit ENaC activity [36,37]. Thus ATP might also be involved in flow-stimulated regulation of ENaC. However this hypothesis requires further investigation.

Achard et al. have shown that pretreatment with colchicine (0.5 mM, 30 min) prevented stretch-induced activation of amiloride-sensitive sodium channels in human B lymphocytes [13]. However, Morimoto et al. defined that flow activation of ENaC is not dependent on intact microtubules [31]. An increase in luminal flow rate in colchicine-treated (10 μM; 1 hr) tubules was associated with an increase in net Na^+ ^absorption and was not different from control CCDs. Awayda and Subramanyam have pretreated *X. laevis *oocytes with Cytochalasin B for 2-5 hrs. rENaC expressed in *X. laevis *oocytes remained insensitive to mechanical perturbations after disruption of the actin cytoskeleton. However, the authors did not observe mechanosensitivity of the channel even at basal conditions [5].

The rationale for these studies comes from our previously published data showing that several small G proteins, including RhoA and Rac1 alter ENaC activity [21-23,38]. These small G proteins are key signal transduction molecules, not only mediating ligand-induced changes to the actin cytoskeleton, but also implicated in numerous cellular processes including vesicle trafficking. Moreover, recent studies reveal that the cytoskeleton is involved in regulation of ENaC and is necessary for small G protein mediated increase of ENaC activity [20]. Yu et al. also observed that in response to a dynamic mechanical environment, increased apical membrane tension, but not pressure, stimulated apical membrane exocytosis and ion transport in bladder umbrella cells. The exocytic response was independent of temperature but required the cytoskeleton and the activity of a nonselective cation channel and the epithelial sodium channel [39]. Consequently, we focused on the flow-mediated regulation of ENaC by the actin cytoskeleton and small GTPase RhoA. Our results suggest that flow activates ENaC overexpressed in CHO cells and, furthermore, that this effect is not mediated by the actin cytoskeleton and RhoA/Rho-kinase signaling pathway. Thus, an exact mechanism of ENaC's activation by mechanical forces requires further investigation

## Materials and methods

### cDNA constructs and cell culture

All chemicals and materials were purchased from either Fisher Scientific or Sigma unless noted otherwise. CHO cells were maintained with standard culture conditions and transfected using Polyfect reagent (Qiagen, Valencia, CA) as described previously [40]. For expression of mouse ENaC in CHO cells, subunit cDNA transfection ratios of 1: 1: 1 were used (0.3 μg of each cDNA per 35 mm dish). To define successfully transfected cells 0.5 μg of green fluorescent protein cDNA (eGFP) was also added to cDNA mix. The expression vectors encoding constitutively active (G14V) or dominant negative (T19N) RhoA were from the UMR cDNA Resource Center http://www.cdna.org. The mammalian expression vectors encoding α-, β- and γ-mouse ENaC have been described previously [41].

### Electrophysiology

Whole-cell macroscopic current recordings of mENaC expressed in CHO cells were made under voltage-clamp conditions using standard methods. In brief, current through ENaC was the inward, amiloride-sensitive Na^+^-current with a bath solution of (in mM) 160 NaCl, 1 CaCl_2_, 2 MgCl_2 _and 10 HEPES (pH 7.4) and a pipette solution of (in mM) 120 CsCl, 5 NaCl, 2 MgCl_2_, 5 EGTA, 10 HEPES, 2.0 ATP and 0.1 GTP (pH 7.4). Current recordings were acquired with an Axopatch 200B (Mol. Devices, Sunnyvale, CA) patch clamp amplifier interfaced via a Digidata 1440A (Mol. Devices) to a PC running the pClamp 10.2 suite of software (Mol. Devices). Cells were clamped to a 40 mV holding potential with voltage ramps (300 ms) from 60 mV down to -100 mV used to elicit current. Flow stimulation was applied by switching ValveBank^®^8 Teflon Perfusion System (AutoMate Scientific, Inc.). ENaC activity is the amiloride-sensitive current density at -80 mV. Whole-cell capacitance, on average 8-10 pF, was compensated. Series resistances, on average 2-5 MΩ, were also compensated.

### Staining with rhodamin-phalloidin

CHO cells were seeded on 12 mm round-shaped coverslips coated with Poly-D-Lysine (BD Bioscience) 24 hrs before experiments and grown to confluence of 70%. Before staining the cells were washed with PBS and fixed with 1.2% paraformaldehyde (Sigma). Then cells were treated with 0,1% Tryton X-100 (Sigma) for 5 min, washed with PBS and incubated with 2 μM rhodamine-phalloidin (Invitrogen) for 15 min at 37°C. Stained cells were mounted on glass slides with Vectashield mounting Medium (Vector Laboratories).

### Microscopy

CHO cells stained with rhodamine-phalloidin were visualized with fluorescence microscope Eclipse E-600 (Nikon) using a 60 × oil objective equipped with a CCD camera (Prinston Instruments). The signal was excited at 546 nm and emission was collected at 590 nm. Fluorescent images were processed with the Metamorph 7.5 software (Molecular Devices) and open source software ImageJ 1.42q http://rsb.info.nih.gov/ij.

### Statistics

All summarized data are reported as means ± SEM. Tau for ENaC inhibition was calculated using a single exponential curve. Data are compared using either the Student's (two-tailed) *t*-test or a one way ANOVA and P < 0.05 is considered significant.

## Competing interests

The authors declare that they have no competing interests.

## Authors' contributions

AVK and DVI carried out the study, participated in its design and coordination and helped to draft the manuscript. AS conceived and designed the experiments, analyzed the data and wrote the paper. All authors read and approved the final manuscript.
